# Investigating Performance, Functional Outcomes, and Patient Autonomy in a Rural Community Hospital: A Real-Life Descriptive Cohort Study of Territorial Intermediate Care

**DOI:** 10.3390/healthcare14121757

**Published:** 2026-06-18

**Authors:** Fabio Del Duca, Luca Casertano, Luca Di Sarra, Arturo Cavaliere, Paola Frati, Gennaro Scialò, Emiliano Cingolani, Aniello Maiese

**Affiliations:** 1 Department of Biomedicine and Prevention, University of Rome “Tor Vergata”, 00133 Rome, Italy; fabio.delduca@uniroma1.it; 2 Unit of Clinical Risk Management, San Camillo-Forlanini Hospital, 00152 Rome, Italyecingolani@scamilloforlanini.rm.it (E.C.); 3 Nursing Management of Intermediate Care Unit, 03100 Frosinone, Italy; 4 Local Health Authority of Frosinone, General Directorate, 03100 Frosinone, Italy; 5 Department of Anatomical, Histological, Forensic and Orthopaedic Sciences, Sapienza University of Rome, Viale Regina Elena 336, 00161 Rome, Italy

**Keywords:** intermediate care, community hospital, clinical risk management, adverse event, performance

## Abstract

**Background/Objectives**: Community hospitals can be a valuable and cost-effective resource for elderly people, especially in rural areas. Their aim is to promote self-reliance, prevent unnecessary hospital admissions, and facilitate rapid recovery after acute illness. The widespread adoption of intermediate care facilities helps alleviate hospital overcrowding by preventing clinical deterioration through advanced and continuous nursing care. An intermediate care unit was established in a rural area of central Italy. This study aims to describe the impact of a community hospital on patients’ functional status from admission to discharge, describing a real-life model. **Methods**: This single-center descriptive study examines trends in the quality of care provided. Data were retrieved from anonymized electronic clinical records. Statistical analyses were performed using descriptive statistics, paired *t*-tests, and Pearson correlation coefficients. **Results**: A total of 532 residents (mean age 80.7 ± 13.2 years; 61% female) were admitted to the community hospital between January 2022 and September 2025. The mean length of stay was 15.2 ± 7.6 days, with a mean improvement in Modified Barthel Index score of 5.24 ± 7.95 (*p* < 0.05). Most patients (81.8%) were discharged home, while 6.0% required hospitalization. No readmissions were recorded in 2025. Clinical risk events occurred only in 1.2% of the total. Nursing specialization increased during the study period, correlating with improved patient outcomes (R = 0.88). **Conclusions**: This descriptive cross-sectional study in a rural nurse-led intermediate care unit found relatively short lengths of stay, high rates of home discharges and modest, but statistically significant, improvements in functional autonomy.

## 1. Introduction

In middle and high-income countries, there has been a steady increase in multimorbidity-associated hospital admissions, largely driven by the growing burden of multiple chronic conditions in an aging population [1,2,3].

This increase has led to the frequent discharge of patients who no longer meet criteria for acute inpatient care, yet remain functionally dependent, with substantial unmet care needs [4]. Such transitions, often characterized by inadequate support in activities of daily living and incomplete medical management, are strongly associated with increased rates of unplanned acute care readmission among older adults with multimorbidity [1,5,6].

Modifiable contributors to high readmission rates—including non-adherence to therapy and delayed detection of clinical decompensation in pre-existing chronic conditions—can be substantially mitigated through coordinated hospital-to-home transitional care [7,8,9].

Transitional care models have demonstrated to improve patient satisfaction and appropriateness of care [1,10,11,12,13]. Hospital-to-home transitional care models typically encompass home-visiting programs [14,15,16], telemonitoring [17,18,19], and nurse-led units.

In rural areas, many post-acute patients require a high level of support that can not be provided within the community, creating a significant challenge for public healthcare organizations [5].

In such cases, intermediate care units represent a specific model of transitional care that plays a central role within the hospital-to-home continuum [20]. These nurse-led facilities deliver structured interventions designed to meet post-acute patients’ needs; as a matter of fact, such patients, although medically stable, still require intensive nursing support and rehabilitation prior to safe discharge home [21].

Although outcome measurement in intermediate care units is currently under active research, evidences in this field remain limited [22]. From a cost perspective, post-discharge costs following acute illness appear to be lower in intermediate care settings; however, findings from cost-effectiveness studies remain inconsistent [23,24].

The predominant model of intermediate care is the community hospital [22]. Such facilities typically comprise general intermediate care units or dedicated post-acute discharge units, operating under a predominantly nurse-led system with substantial clinical support from general practitioners (GPs) [25], who frequently retain responsibility for patient admission, clinical management, and discharge decisions.

In Italy [26], where general intermediate care units (ImCUs) remain relatively uncommon, specialized intermediate care models—particularly those focusing on nurse-led rehabilitation and monitoring—have demonstrated favorable outcomes.

Given the heterogeneity of intermediate care models and the limited evidence from rural settings, this study aimed to evaluate the effectiveness and performance of a nurse-led intermediate care model in Frosinone, a rural area of Lazio Region, Italy.

The Province of Frosinone has approximately 500,000 inhabitants, mostly scattered in rural areas, therefore with limited access to specialized acute care facilities.

The present study aimed to describe, in a structured and measurable manner, the real-life performance of a nurse-led intermediate care unit in a rural Italian setting.

Our main goal was to determine whether admission to this rural community hospital, between 2022 and 2025, was associated with statistically significant improvements in patient functional autonomy (measured as Modified Barthel Index change [27], Δ-MBI), high rates of home discharge, and reduced 30-day hospital readmissions rates, while preserving an acceptable safety profile.

Secondary objectives were to examine temporal trends in discharge destination, length of stay, and level of nursing specialization over the four-year study period, and to explore the association between high nursing specialization and both improved functional outcomes and appropriateness of care.

## 2. Materials and Methods

### 2.1. Study Design and Data Collection

This single-center, descriptive observational study was conducted at an intermediate care facility affiliated with the Local Health Authority (ASL) of Frosinone, Italy. Data were retrieved from anonymized electronic clinical records.

This study was reported in accordance with the Strengthening the Reporting of Observational Studies in Epidemiology (STROBE) guidelines (Supplementary File S1).

The dataset comprised all admissions from 1 January 2022 to 30 September 2025. Eligibility criteria included: age ≥ 18 years, clinical stability following acute hospitalization, need for ongoing nursing support, and ineligibility for acute hospital readmission. The capacity to engage in rehabilitation was clinically assessed; use of assistive devices during functional assessments was permitted. Patients were excluded if clinical records were incomplete or if transfer for acute deterioration occurred within 24 h of admission (*n* = 15; 2.7% of all referrals)—because they reflect rapid clinical instability requiring acute hospital-level intervention, consistent with protocol eligibility requirements.

All consecutive admissions meeting these inclusion criteria were enrolled; no refusals were recorded. Data extraction covered demographic variables (age, sex, residence), clinical parameters (admission diagnoses, length of stay [LOS], discharge destination), functional assessments, and adverse events. All data were aggregated and de-identified prior to analysis to maintain confidentiality.

### 2.2. Setting and Organizational Model

The facility operates as an intermediate care unit, providing low-intensity medical interventions alongside continuous, high-level nursing care. It serves patients of all age groups who are medically stable yet have significant dependency in activities of daily living, rendering them unsuitable for acute hospital settings or immediate home discharge.

Admissions typically follow acute hospitalizations when patients require completion of therapeutic regimens, ongoing monitoring, or social support that cannot feasibly be provided at home. Access is governed by a multidisciplinary team comprising the referring hospital physician, general practitioner (GP), or specialist, together with a nurse coordinator or case manager, who jointly determine eligibility, based on clinical stability and care needs.

Care is nurse-led, with registered nurses serving as primary caregivers, assisted by healthcare assistants. Medical oversight is provided through on-call GPs or consulting hospital physicians, who provide specialist input as needed. The facility offers 24 h nursing coverage, ensures seamless integration with hospital and community services, and employs individualized nursing care plans (Individual Support Plan, ISP) to guide clinical management. Core objectives include enhancing patient autonomy, preventing complications, optimizing discharge to home or appropriate long-term care facilities, and reducing unnecessary acute hospitalization.

Clinical eligibility is assessed using the Modified Barthel Index. This model aligns with national guidelines for intermediate care, emphasizing resource efficiency and patient-centered outcomes.

### 2.3. Assessment of Patient Needs

Patient functional status was evaluated using the Modified Barthel Index (MBI), a validated multidimensional tool that quantifies independence in activities of daily living across ten functional domains: feeding, bathing, grooming, dressing, bowel control, bladder control, toilet use, bed-to-chair transfers, mobility on level surfaces, and stairs climbing. Each domain is scored according to the level of assistance required, yielding a total score from 0 (complete dependence) to 100 (full independence). Scores are conventionally stratified into five dependency categories: total dependence (0–20), severe dependence (21–60), moderate dependence (61–90), slight dependence (91–99), and independence (100).

MBI scores were recorded numerically at admission (MBI IN) and discharge (MBI OUT) to enable precise evaluation rather than categorical classification. The change in MBI score (Δ-MBI = MBI OUT − MBI IN) served as a proxy for functional improvements and care efficacy, and was examined in relation to resource utilization and length of stay.

### 2.4. Statistical Analysis

Analyzed variables included patient demographics, nursing interventions, and healthcare outcomes. Data were aggregated and de-identified prior to analysis. Descriptive statistics included frequencies, percentages, means (±standard deviation, SD), medians, interquartile ranges (IQR), and ranges as appropriate. Pearson’s correlation coefficient (r) was calculated to examine relationships between variables such as age, LOS, MBI scores, and outcomes. Paired *t*-tests evaluated changes in MBI scores pre- and post-admission, with significance set at *p* < 0.05. All analyses were performed using GraphPad Prism version 10.6.1 (GraphPad Software, Boston, MA, USA).

### 2.5. Biases

The progressive shift in case-mix toward a higher proportion of nursing-oriented and medium-to-high intensity care admissions over the four-year period introduces potential confounding bias [28], as changes in patient characteristics may have influenced the observed outcomes. Given that data were available in aggregated form only, multivariable adjustment for diagnosis, age, baseline functional status, and temporal trends was not feasible. Furthermore, changes in Modified Barthel Index scores are susceptible to regression-to-the-mean bias, particularly in patients with low baseline scores [29]. Finally, the absence of socioeconomic, insurance, and equity data limits the generalizability of findings across different patient subgroups [30].

## 3. Results

### 3.1. Patient Characteristics

A total of 532 patients were included in the analysis (Table 1): 146 admitted in 2022, 166 in 2023, 161 in 2024, and 79 in 2025. The mean (±SD) age of the overall cohort was 80.7 ± 13.2 years (95% CI: 79.65–81.75) with mean annual age ranging from 77.0 to 82.0 years across the study period. The sex distribution comprised 322 females and 210 males.

Mean length of stay was 15.2 ± 7.6 days, with 1.3% of stays lasting 30 days or longer. The mean improvement in Modified Barthel Index score from admission to discharge was 5.24 ± 7.95 (paired *t*-test, *p* < 0.05, 95% CI [4.56, 5.92]). Discharge to home was achieved in 81.8% of cases, while 6.0% of patients required hospitalization.

The most frequent primary admission diagnoses were: nursing care for stabilization and clinical monitoring (24.1%), motor reactivation with recovery of self-sufficiency and clinical monitoring (16.4%), motor reactivation with mood monitoring (9.4%), medium-to-high intensity health care (8.3%), motor rehabilitation (6.8%), and clinical monitoring with pharmacological therapy adjustment (6.4%); femoral fracture accounted for 2.6% of admissions. Thirty-day hospital readmission rate decreased progressively from 2022 to 2025, with an initial rate of 3.38% for the same diagnosis; no readmissions were recorded in 2025.

### 3.2. Pearson R Correlation in Aggregates Values

Age showed weak positive correlations with length of stay (r = 0.12), mortality (r = 0.09), and 30-day rehospitalization (r = 0.09), and moderate negative correlations with both baseline MBI score (r = −0.37) and discharge MBI score (r = −0.38). Distance from home had negligible correlations with all variables (highest |r| = 0.09 for length of stay). Length of stay correlated positively with Δ-MBI (r = 0.41) and negatively with baseline MBI score (r = −0.14). Mortality showed weak negative correlations with length of stay (r = −0.11) and all MBI-related measures. Thirty-day rehospitalization was negatively correlated with all MBI measures, with the strongest correlation observed for discharge MBI score (r = −0.24). Given the limited number of annual data points (*n* = 4), correlation analyses at aggregate level are statistically unreliable and susceptible to wide confidence intervals and ecological fallacy; therefore, temporal trends are presented descriptively only, without inferential testing.

During the study period (2022–2025), the proportion of admissions motivated by predominantly nursing-oriented diagnoses increased progressively. This trend coincided with improvements in mean Δ-MBI, higher home discharge rates, and a steady decline in 30-day readmission rates, reaching zero in 2025.

Although these concurrent trends are consistent with a potential contribution of growing nursing specialization to support functional recovery and care appropriateness, the small number of annual data points precludes any formal statistical assessment of association at the aggregate level.

**Figure 1:**
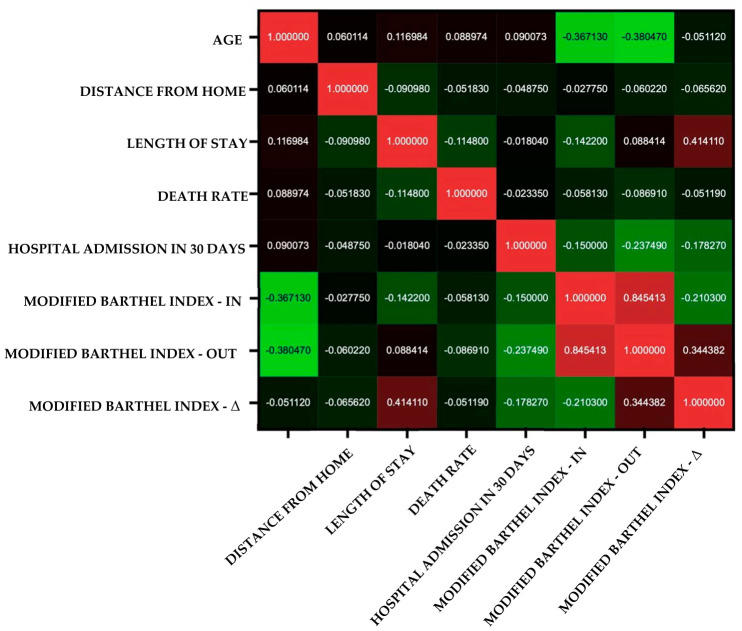
Analysis of r-Pearson. Significant improvements in Modified Barthel Index scores are associated with increased daily autonomy. Abbreviation: MODIFIED BARTHEL INDEX—Δ: difference in MBI value at the admission (MBI-IN) and MBI at the discharge (MBI-out).

Admission and discharge MBI scores were strongly correlated (r = 0.85). Δ-MBI correlated positively with length of stay (r = 0.41) and discharge MBI score (r = 0.34), and negatively with baseline MBI score (r = −0.21) (Figure 1).

Given the strong correlation between length of stay and Δ-MBI (r = 0.41), this relationship is further examined across calendar years (Table 2).

Data from four consecutive years (2022–2025) were analyzed. The mean Δ-MBI ranged from 3.86 to 8.19, with the highest value recorded in 2025. Mean length of stay varied between 14.32 and 16.53 days across the study period. All within-year paired comparisons yielded statistically significant differences (*p* < 0.0001 for each year).

### 3.3. Performance

**Figure 2:**
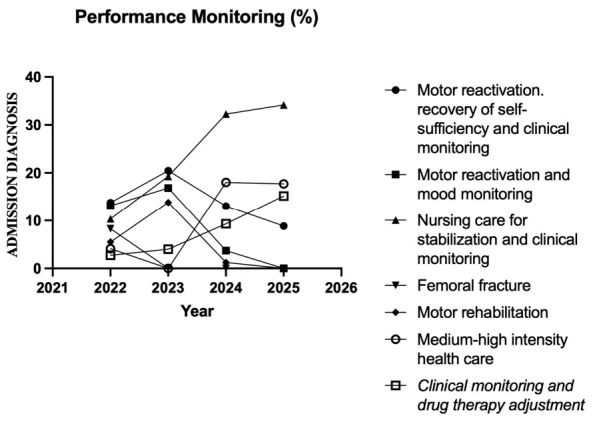
It is shown here the trend of admission diagnoses. The proportion of nursing-oriented admissions increased over time, reflecting growing clinical expertise and improved referral appropriateness. Proportion of total admissions (%) by primary admission diagnosis category per year. Percentages are calculated within each calendar year and summed to 100%.

The cohort encompassed patients with a range of admission diagnoses distributed across predefined clinical categories. The proportion admitted for “motor reactivation, recovery of self-sufficiency, and clinical monitoring” ranged from 8.86% to 20.48% across the study years (Figure 2).

Cases admitted for “motor reactivation and mood monitoring” varied between 0% and 16.86%. Patients requiring “nursing care for stabilization and clinical monitoring” represented between 10.34% and 34.17% of the sample. Admissions for femoral fracture decreased from 8.27% in 2022 to 0% in subsequent years. “Motor rehabilitation” cases ranged from 0% to 13.82%, while those classified under “medium-high intensity health care” varied from 0% to 18.01%. The percentage of patients admitted for “clinical monitoring and drug therapy adjustment” ranged between 2.74% and 15.19%.

The proportion of patients admitted for “motor reactivation, recovery of self-sufficiency, and clinical monitoring” increased from 13.8% initially to 20.5%, before stabilizing between 8.9% and 16.4% in subsequent periods, reflecting growing clinical expertise in managing these patients.

Mean MBI scores at admission (MBI IN) and discharge (MBI OUT) were compared across diagnostic categories. Statistically significant improvements were observed in patients admitted for “motor reactivation, recovery of self-sufficiency, and clinical monitoring” (MBI IN 75.43 vs. MBI OUT 80.67; *p* = 0.004), “motor reactivation and mood monitoring” (73.38 vs. 80.43; *p* < 0.0001), and “nursing care for stabilization and clinical monitoring” (71.43 vs. 77.14; *p* = 0.02).

No statistically significant improvements were observed for “femoral fracture” (72.05 vs. 78.33; *p* = 0.06), “motor rehabilitation” (73.53 vs. 78.14; *p* = 0.08), “medium-high intensity health care” (86.67 vs. 90.19; *p* = 0.24), or “clinical monitoring and drug therapy adjustment” (79.95 vs. 85.38; *p* = 0.15) (Table 3).

Overall, the total cohort showed a statistically significant improvement in MBI scores from admission to discharge (75.62 vs. 81.29; *p* < 0.001; r = 0.88).

### 3.4. Risk Management Assessment

During 2023–2025, the only reported clinical risk events were pressure ulcers, with annual rates ranging from 0.61% to 2.48% and an overall incidence of 1.12%. No cases of physical restraint, accidental falls, or other adverse events were recorded (Table 4).

## 4. Discussion

Intermediate care facilities are increasingly recognized as effective models of community-based healthcare with cost-reduction potential [23,26,31]; however, the evidence base for clinical outcomes remains limited.

The clinical effectiveness of intermediate care remains debated, as the available literature is often limited in scope or methodological rigor. Nevertheless, the prevailing trend in the international literature indicates a generally favorable outlook for the implementation of intermediate care [32].

The present study describes a systematic monitoring of functional status changes, demographic variables, and clinical parameters in a rural model of intermediate care.

In this single-center analysis, 532 patients admitted to a nurse-led inpatient unit between 2022 and 2025 were predominantly elderly (mean age 80.7 years), with a mean length of stay of 15.2 days. Mean patient age is consistent with values reported in the literature, whereas the length of stay is notably shorter than the 22.4 [33] to 25 days [34] reported for comparable settings.

Home discharge occurred in over 80% of cases, and hospital readmission within 30 days was infrequent (6.01%). Published data report a mean 30-day post-discharge hospitalization rate of 11.1% among elderly patients admitted to intermediate care settings [35].

The low rehospitalization rate may reflect the individualized, patient-centered care delivered by the nurse-led model [32]. This approach enables optimized resource allocation by reducing hospital admissions, shortening lengths of stay, and supporting safe discharge home through personalized rehabilitation and continuity of care [33,34,35,36,37,38,39,40,41,42,43,44,45,46,47].

Critically, it enhances patient autonomy and facilitates safe home discharge—outcomes achieved through personalized rehabilitation and continuity of care [36,37,38,39,40,41,42,43,44,45,46,47,48,49,50].

In contrast to some of the existing literature [51], the present study suggests a possible influence on both discharge destination and reduction in functional dependence. This model uniquely bridges secondary and primary care gaps, serving as a flexible alternative to both.

Longitudinal observational data suggest possible sustained benefits, with lower 30-day readmission rates among patients discharged under this model. Enhanced discharge preparedness—validated through patient competency assessments—appears to drive these clinical and economic efficiencies [51].

In this study, functional status of patients was investigated using the Modified Barthel Index. Increase in Modified Barthel Index (MBI) before and after discharge from a community hospital was described as correlated.

Overall, however, functional independence measured by the Modified Barthel Index improved significantly (*p* < 0.001). Improvements were consistent across the four-year period, with Δ-MBI positively correlated with length of stay (r = 0.41).

Admissions with predominantly nursing-driven diagnoses were statistically associated with overall functional improvement. Over the four-year observation period, the proportion of such cases—particularly “nursing care for stabilization and clinical monitoring” and “motor reactivation with recovery of self-sufficiency and clinical monitoring”—increased progressively, suggesting consolidation of the nurse-led care model and enhanced capacity to manage complex, high-dependency patients without acute medical supervision.

These categories show consistent and clinically meaningful MBI gains (*p* < 0.05), underscoring the prognostic impact of structured, continuous nursing interventions on patient autonomy.

One possible explanation is the unit’s growing specialization in managing patients who require primarily nursing care and clinical monitoring. Over the study period, the proportion of such admissions increased, reflecting more appropriate referrals, but this correlation requires further evidence, even if studies of ICUs are promising.

The temporal increase in these admissions parallels the observed rise in discharge-to-home rates and the low incidence of adverse events, supporting the role of targeted nursing care pathways in improving functional outcomes while maintaining a safe care environment [32].

In the present study, adverse events were rare, limited to pressure ulcers in 1.1% of cases, and no falls or physical restraints use were recorded. These findings suggest that the nurse-led intermediate care model can safely support functional recovery and timely discharge in clinically stable, high-dependency patients.

Follow-up telephone assessments, conducted as specified in the service charter, indicated a 30-day readmission rate of 1.31%.

Healthcare costs are a critical consideration in sustaining public health systems in high-income countries. Comparable intermediate care models have reported mean hospital stays of approximately 29.4 days, reflecting the higher intensity and resource use associated with more complex patient profiles. Although this level of care initially raises health expenditures, sensitivity analyses suggest that length of stay is a key cost driver, and reductions in this metric could substantially offset overall expenditure [23].

In this study, pharmaceutical expenditure is presented only as a descriptive benchmark for international comparisons. It appeared modest compared with earlier reports from similar units. However, these data should not be interpreted as evidence of cost-saving or cost-effectiveness. Future studies should examine whether pharmaceutical costs decline over time in this setting.

This four-year study shows that intermediate care, delivered with specialized nursing expertise and supported by medical consultations and rehabilitative services, appears to be safe and effective. This model could lead to improved prescribing appropriateness, shorter lengths of stay, lower 30-day readmission rates, fewer adverse events, and a reduction in overall costs per patient [52]. Taken together, these results suggest that structured intermediate care can provide better outcomes and greater efficiency than previously reported models [53].

### Limitations

The progressive shift toward a higher proportion of nursing-oriented and medium-to-high intensity care admissions over the study period likely reflects both improved referral appropriateness and adaptation of the model to more complex, clinically stable post-acute patients.

This change in case-mix represents an important potential confounder that precludes causal attribution of observed outcome improvements to any single factor, including increased nursing expertise. Formal multivariable adjustment for diagnosis, age, baseline functional status, and temporal effects would be required to disentangle these influences but was not feasible given the aggregated nature of the available data and the descriptive aims of the study.

The limited administrative data available did not allow for a formal health economic analysis (e.g., adjustment for inflation, per-patient costs, or comparison with standard care). Accordingly, all references to pharmaceutical expenditure are purely descriptive, and no claims of cost-effectiveness or cost-saving are made. The mean pharmaceutical cost per patient is included solely as a potentially informative benchmark for the international scientific community.

The change in Modified Barthel Index (Δ-MBI) is susceptible to regression-to-the-mean bias (particularly in patients with low baseline scores) and ceiling effects, both of which may contribute to observed improvements independent of the model’s effect. Stratified analyses or reliable change indices were not feasible in this descriptive study.

No socioeconomic, insurance, or equity data were collected. Consequently, the study cannot assess equitable benefit distribution or whether outcomes favored higher-functioning or younger patients—an important gap for future equity-focused research.

The single-center, single-arm design precludes causal inference and adjustment for unmeasured confounders.

## 5. Conclusions

This descriptive cross-sectional study found that the nurse-led intermediate care model in a rural community hospital was associated with relatively short lengths of stay and high rates of discharge to home. Functional autonomy, as measured by the Modified Barthel Index, showed a modest but statistically significant improvement between admission and discharge. Adverse events were rare, limited almost exclusively to pressure ulcers.

The progressive increase in admissions motivated by nursing-oriented diagnoses coincided with improved functional outcomes and greater appropriateness of care. Although these findings are descriptive and derive from a single-center experience, they suggest that this nurse-led intermediate care model may represent a safe and effective component within the transitional care continuum, particularly in rural settings where access to acute hospital services is limited.

Further multicenter, controlled studies are needed to confirm these findings and to evaluate the long-term impact of nurse-led intermediate care on patient outcomes and healthcare utilization.

## Figures and Tables

**Table 1:** Descriptive analysis of the study cohort over the period 2022–2025.

Characteristic	2022	2023	2024	2025	Total
Residents—no.	146	166	161	79	532
Age—yr	82 ± 11.7	80.83 ± 15.12	80.35 ± 12.21	77.03 ± 13.20	80.68 ± 13.22
F:M	89:57	99:67	103:52	41:34	324:208
Length of stay—days	16.53 ± 7.74	14.32 ± 6.77	14.72 ± 7.55	15.36 ± 8.41	15.22 ± 7.56
Length of stay ≥ 30 days—no. (%)	2.32%	0%	1.20%	1.26%	1.31%
Δ-MBI [MBI IN—MBI OUT]	5.76 ± 8.90	3.86 ± 7.24	4.62 ± 6.43	8.19 ± 9.38	5.24 ± 7.95
Discharge home (%)	77.03%	82.53%	83.85%	84.81%	81.8%
Hospitalization	4.82%	7.23%	5.59%	6.33%	6.01%
Readmission in hospital within 30 days	3.38%	1.82	0.62%	0%	1.31%
Pharmaceutical expenditure (€)	15,304.65	16,667.50	19,372.26	8900.53	60,244.94 (109.53 per patient)
ADMISSION DIAGNOSIS—no. (%)					
Motor reactivation, recovery of self-sufficiency and clinical monitoring	13.79%	20.48%	13.04%	8.86%	16.35%
Motor reactivation and mood monitoring	13.10%	16.86%	3.72%	0%	9.39%
Nursing care for stabilization and clinical monitoring	10.34%	19.27%	32.29%	34.17%	24.06%
Femoral fracture	8.27%	0%	0%	0%	2.63%
Motor rehabilitation	5.51%	13.82%	1.24%	0%	6.76%
Medium-high intensity health care	4.13%	0%	18.01%	17.72%	8.25%
Clinical monitoring and drug therapy adjustment	2.74%	4%	9.32%	15.19%	6.37%

**Table 2:** Yearly comparison of Δ-MBI and length of stay (2022–2025).

Year	Δ-MBI	Length of Stay (Days)	*p*-Value *
2022	5.76 ± 8.90	16.53 ± 7.74	<0.0001
2023	3.86 ± 7.24	14.32 ± 6.77	<0.0001
2024	4.62 ± 6.43	14.72 ± 7.55	<0.0001
2025	8.19 ± 9.38	15.36 ± 8.41	<0.0001

* *p*-values were derived from paired *t*-tests comparing Modified Barthel Index scores at admission versus discharge within each calendar year.

**Table 3:** Association between intermediate care admission and functional outcomes according to primary nursing diagnosis.

Admission Diagnosis—No. (%)	Mean MBI in	Mean MBI out	*p* Value
Motor reactivation, recovery of self-sufficiency and clinical monitoring	75.43	80.67	0.004 (*p* < 0.01)
Motor reactivation and mood monitoring	73.38	80.43	<0.0001
Nursing care for stabilization and clinical monitoring	71.43	77.14	0.02 (*p* < 0.05)
Femoral fracture	72.05	78.33	0.06 (*p* > 0.05)
Motor rehabilitation	73.53	78.14	0.08 (*p* > 0.05)
Medium-high intensity health care	86.67	90.19	0.24 (*p* > 0.05)
Clinical monitoring and drug therapy adjustment	79.95	85.38	0.15 (*p* > 0.05)
TOTAL	75.62	81.29	<0.001 (r = 0.88)

**Table 4:** Events recorded by the Clinical Risk Management Unit of Local Health Authority of Frosinone.

Clinical Risk Events	2023	2024	2025	Total
Pressure Ulcers	0.61%	2.48%	1.23%	1.12%
Restraint	0	0	0	0
Fall, accidental	0	0	0	0
Adverse events	0	0	0	0

## Data Availability

The datasets are not publicly available due to privacy and ethical restrictions (containing sensitive health data). However, they are available upon reasonable request from the corresponding author.

## Supplementary Materials

The following supporting information can be downloaded at: https://www.mdpi.com/article/10.3390/healthcare14121757/s1, File S1: STROBE Statement—Checklist of items that should be included in reports of cross-sectional studies.

## Abbreviations

The following abbreviations are used in this manuscript:

MBI	Modified Barthel Index
MBI-IN	Modified Barthel Index at the admission
MBI-OUT	Modified Barthel Index at the discharge
MBI-Δ	Modified Barthel Index increase/decrease
LOS	Length of stay
