# Supplementary material for: Investigating Performance, Functional Outcomes, and Patient Autonomy in a Rural Community Hospital: A Real-Life Descriptive Cohort Study of Territorial Intermediate Care
Source: Healthcare (Basel). 2026 Jun 18;14(12):1757. doi: 10.3390/healthcare14121757 (PMC13299622; doi:10.3390/healthcare14121757)
Supplement: Supplementary file 1 [file healthcare-14-01757-s001.zip › healthcare-4291323-supplementary.pdf]

STROBE Statement—Checklist of items that should be included in reports of ***cross-sectional studies***

	Item No	Recommendation
**Title and abstract**	1	(*a*) Indicate the study's design with a commonly used term in the title or the abstract
		(*b*) Provide in the abstract an informative and balanced summary of what was done and what was found
		Lines 2-4
		Lines 19-38
**Introduction**		
Background/rationale	2	Explain the scientific background and rationale for the investigation being reported
		Lines 43 – 66: Background
		Lines 67 – 82: Rationale
Objectives	3	State specific objectives, including any prespecified hypotheses
		Lines 86 - 96
**Methods**		
Study design	4	Present key elements of study design early in the paper
		Lines 99 – 100
Setting	5	Describe the setting, locations, and relevant dates, including periods of recruitment, exposure, follow-up, and data collection
		Lines 120 - 142
Participants	6	(*a*) Give the eligibility criteria, and the sources and methods of selection of participants
		Lines 106 - 114
Variables	7	Clearly define all outcomes, exposures, predictors, potential confounders, and effect modifiers. Give diagnostic criteria, if applicable
		Lines 138- 178
Data sources/ measurement	8*	For each variable of interest, give sources of data and details of methods of assessment (measurement). Describe comparability of assessment methods if there is more than one group (N/A)
		Lines 122-125 and Lines 166-168
Bias	9	Describe any efforts to address potential sources of bias
		Lines 189-200
Study size	10	Explain how the study size was arrived at
		Lines 138-141
Quantitative variables	11	Explain how quantitative variables were handled in the analyses. If applicable, describe which groupings were chosen and why
Statistical methods	12	(*a*) Describe all statistical methods, including those used to control for confounding
		(*b*) Describe any methods used to examine subgroups and interactions
		(*c*) Explain how missing data were addressed
		(*d*) If applicable, describe analytical methods taking account of sampling strategy
		(*e*) Describe any sensitivity analyses
		Lines 180-188
**Results**		
Participants	13*	(a) Report numbers of individuals at each stage of study—eg numbers potentially eligible, examined for eligibility, confirmed eligible, included in the study,

		completing follow-up, and analysed
		(b) Give reasons for non-participation at each stage
		(c) Consider use of a flow diagram
		Not applicable: In this rural setting, patients who did not meet the eligibility criteria for intermediate care (see exclusion criteria) were never admitted to the unit; they were either transferred directly to other acute hospitals or discharged home. This was not available.
Descriptive data	14*	(a) Give characteristics of study participants (eg demographic, clinical, social) and information on exposures and potential confounders
		(b) Indicate number of participants with missing data for each variable of interest
		Lines 203-222 and Table 1.
Outcome data	15*	Report numbers of outcome events or summary measures
		Lines 223-261
Main results	16	(*a*) Give unadjusted estimates and, if applicable, confounder-adjusted estimates and their precision (eg, 95% confidence interval). Make clear which confounders were adjusted for and why they were included
		(*b*) Report category boundaries when continuous variables were categorized
		(*c*) If relevant, consider translating estimates of relative risk into absolute risk for a meaningful time
		Lines 203-2011
Other analyses	17	Report other analyses done—eg analyses of subgroups and interactions, and sensitivity analyses
		Lines 224-236
**Discussion**		
Key results	18	Summarise key results with reference to study objectives
		Lines 306-311
Limitations	19	Discuss limitations of the study, taking into account sources of potential bias or imprecision. Discuss both direction and magnitude of any potential bias
		Lines 393 -317
Interpretation	20	Give a cautious overall interpretation of results considering objectives, limitations, multiplicity of analyses, results from similar studies, and other relevant evidence
		Lines 345 - 377
Generalisability	21	Discuss the generalisability (external validity) of the study results
		Lines 385 - 391
**Other information**		
Funding	22	Give the source of funding and the role of the funders for the present study and, if applicable, for the original study on which the present article is based
		Line 439

*Give information separately for exposed and unexposed groups.

Note: An Explanation and Elaboration article discusses each checklist item and gives methodological background and published examples of transparent reporting. The STROBE checklist is best used in conjunction with this article (freely available on the Web sites of PLoS Medicine at http://www.plosmedicine.org/, Annals of Internal Medicine at http://www.annals.org/, and Epidemiology at http://www.epidem.com/). Information on the STROBE Initiative is available at www.strobe-statement.org.
